# Laminin-Coated Poly(Methyl Methacrylate) (PMMA) Nanofiber Scaffold Facilitates the Enrichment of Skeletal Muscle Myoblast Population

**DOI:** 10.3390/ijms18112242

**Published:** 2017-10-30

**Authors:** Nor Kamalia Zahari, Ruszymah Binti Haji Idrus, Shiplu Roy Chowdhury

**Affiliations:** 1 Tissue Engineering Centre, Universiti Kebangsaan Malaysia Medical Centre, Cheras 56000, Kuala Lumpur, Malaysia; kamalia_122@yahoo.com (N.K.Z.); ruszyidrus@gmail.com (R.B.H.I.); 2 Department of Physiology, Faculty of Medicine, Universiti Kebangsaan Malaysia Medical Centre, Cheras 56000, Kuala Lumpur, Malaysia

**Keywords:** myoblast, fibroblast, skeletal muscle, electrospinning, poly(methyl methacrylate), laminin, collagen, proliferation, migration

## Abstract

Myoblasts, the contractile cells of skeletal muscle, have been invaluable for fundamental studies of muscle development and clinical applications for muscle loss. A major limitation to the myoblast-based therapeutic approach is contamination with non-contractile fibroblasts, which overgrow during cell expansion. To overcome these limitations, this study was carried out to establish a 3D culture environment using nanofiber scaffolds to enrich the myoblast population during construct formation. Poly(methyl methacrylate) (PMMA) nanofiber (PM) scaffolds were fabricated using electrospinning techniques and coated with extracellular matrix (ECM) proteins, such as collagen or laminin, in the presence or absence of genipin. A mixed population of myoblasts and fibroblasts was isolated from human skeletal muscle tissues and cultured on plain surfaces, as well as coated and non-coated PM scaffolds. PMMA can produce smooth fibers with an average diameter of 360 ± 50 nm. Adsorption of collagen and laminin on PM scaffolds is significantly enhanced in the presence of genipin, which introduces roughness to the nanofiber surface without affecting fiber diameter and mechanical properties. It was also demonstrated that laminin-coated PM scaffolds significantly enhance myoblast proliferation (0.0081 ± 0.0007 h^−1^) and migration (0.26 ± 0.04 μm/min), while collagen-coated PM scaffolds favors fibroblasts proliferation (0.0097 ± 0.0009 h^−1^) and migration (0.23 ± 0.03 μm/min). Consequently, the myoblast population was enriched on laminin-coated PM scaffolds throughout the culture process. Therefore, laminin coating of nanofiber scaffolds could be a potential scaffold for the development of a tissue-engineered muscle substitute.

## 1. Introduction

Numerous natural and synthetic materials, either biodegradable or permanent, have been used as 3D scaffolds for the development of tissue substitutes [1,2]. Generally, these scaffolds mimic the physiological environment of the native tissue, and support cell proliferation, migration, and differentiation [3]. These scaffolds temporarily provide an architecture for seeded cells, which allows cells to produce their extracellular matrix (ECM) to regenerate tissue with the structural integrity and biomechanical properties of native tissue [4,5]. Recently, electrospun nanofiber scaffolds have received tremendous attention in tissue engineering applications, as they mimic the properties of the ECM and provide a suitable environment for cellular adhesion, proliferation, migration, and differentiation, which has contributed to the development of various tissue substitutes [6,7]. The electrospun nanofiber scaffold is highly porous and provides a high surface area to volume ratio and mechanical strength that allows for nutrient transport; these fibers possess structural homology with the ECM [8,9]. Natural, synthetic or composite materials have been used to fabricate nanofiber scaffolds via electrospinning. Poly(methyl methacrylate) (PMMA) is a well-known biocompatible synthetic polymer, generally used for hard tissue repair or regeneration. It has also been used, separately or in combination with another polymer, to fabricate electrospun nanofiber scaffolds for tissue engineering applications [10,11,12,13,14,15].

Skeletal muscle loss commonly occurs with trauma and degenerative muscle diseases, which presents a significant challenge in primary care; complete regeneration is compromised due to the development of fibrotic tissue. Tissue engineering provides an alternative approach to repair lost or damaged muscle tissue. Several studies have demonstrated the effectiveness of electrospun nanofiber scaffolds in the development of skeletal muscle tissue substitutes [16]. Although electrospun nanofiber scaffolds offer many advantages for use as tissue substitutes, inert polymeric materials such as PMMA lack active binding sites for cells. Thus, attempts have been made to functionalize nanofiber scaffolds by immobilizing ECM proteins using functional peptides, with the aim to improve the regeneration potential of the scaffolds for tissue-specific applications [17].

Over the past decades, myoblasts, the contractile cells of skeletal muscle, have been tested for their applications in cell and tissue therapy, and as a gene delivery system to regenerate muscle loss [18]. A major limitation in the myoblast-based therapeutic approach is obtaining large quantities of pure myoblasts. Myoblasts isolated from muscle tissue are contaminated with non-contractile fibroblasts, which grow faster during cell expansion in vitro. Previous studies have tested methods to enrich the myoblast population by culturing cells on a laminin-coated surface [19]. However, to produce muscle tissue substitutes, the enrichment of myoblasts needs to be achieved in a 3D culture environment.

Thus, this study aimed to fabricate an electrospun nanofiber mesh using PMMA, coated with laminin or collagen, to regulate the population of myoblasts and fibroblasts. The physicochemical properties of the coated and non-coated nanofiber (PM) scaffolds were characterized. Then, a mixed population of human myoblasts and fibroblasts was cultured on the coated and non-coated PM scaffolds to evaluate cell morphology, proliferation and migration, and the potential of enriching the myoblast population.

## 2. Results

### 2.1. Collagen and Laminin Coating

**Figure 1:**
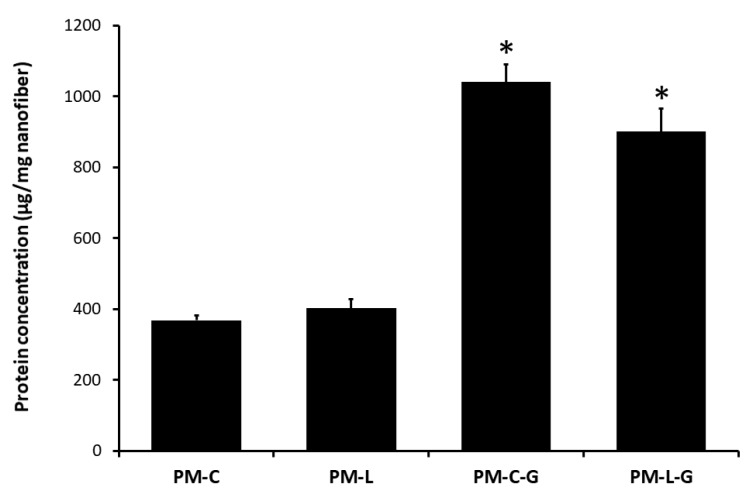
Collagen (C) and laminin (L) adsorption on the poly(methyl methacrylate) (PMMA) nanofiber (PM) scaffold, with and without genipin (G). All groups have been normalized to the PM scaffolds. * Indicates a significant difference for collagen-coated PM scaffolds with genipin (PM-C-G) and laminin-coated PM scaffolds with genipin (PM-L-G) vs. the respective control coating.

Laminin (L) and collagen (C), with or without genipin (G), were used to coat the poly(methyl methacrylate) (PMMA) nanofiber (PM) scaffolds. The results show that cross-linking with genipin enhanced the deposition of laminin and collagen on the PM scaffolds. As shown in Figure 1, the amounts of protein on the collagen-coated PM scaffolds with genipin (PM-C-G; 1040 ± 51 μg/mg of the scaffold) and laminin-coated PM scaffolds with genipin (PM-L-G; 900 ± 65 μg/mg of the scaffold) were approximately three and two times higher compared to those on collagen-coated PM scaffolds without genipin (PM-C) and laminin-coated PM scaffolds without genipin (PM-L) scaffolds, respectively. Thus, considering their superior protein adsorption, PM-C-G and PM-L-G scaffolds were used for subsequent experiments.

### 2.2. Physicochemical Properties of Nanofiber Scaffolds

**Figure 2:**
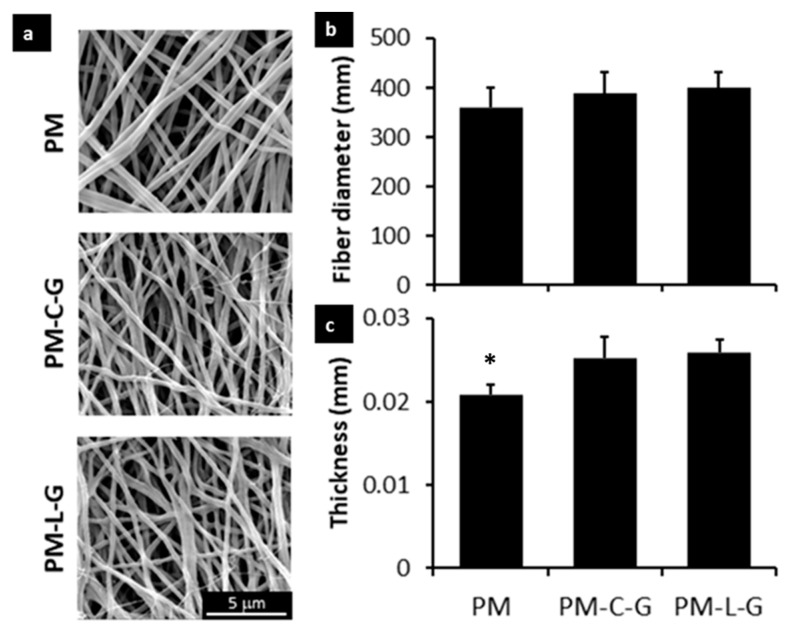
Scanning electron micrographs of (**a**) PM, PM-C-G, and PM-L-G. Bar graphs illustrate the (**b**) average fiber diameter and (**c**) thickness of the PM nanofibers. The coated PM fibers had a rougher surface compared to non-coated PMMA. This confirmed that collagen and laminin had been successfully applied as coatings on the surface. However, there was not a significant difference in fiber diameter (*n* = 90 per fiber type). * indicates significantly lower thickness of PM scaffolds compared to PM-C-G and PM-L-G scaffolds.

**Figure 3:**
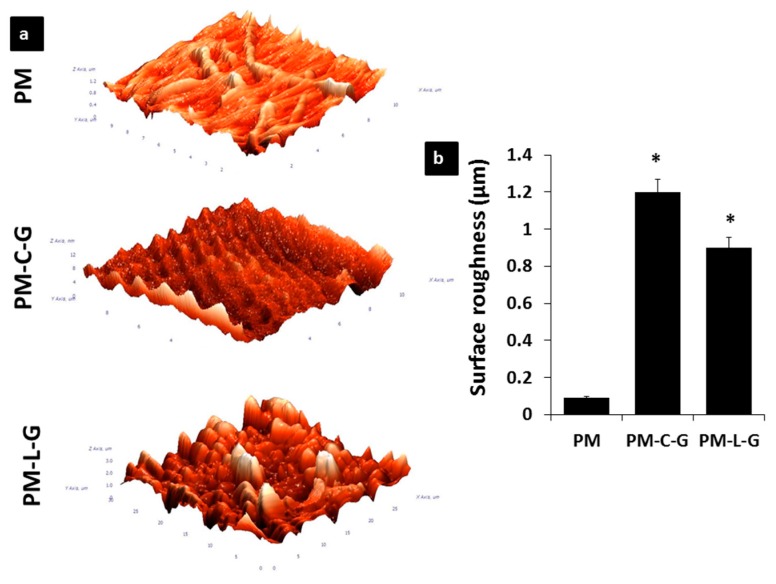
(**a**)Atomic Force Microscopy images of PM, PM-C-G, and PM-L-G; (**b**) The bar graph illustrates the surface roughness of nanofiber scaffolds. There was a significant difference in surface roughness of coated PMMA compared to non-coated PM. * indicates significantly higher surface roughness of PM-C-G and PM-L-G compared to PM scaffolds.

The PM, PM-C-G, and PM-L-G scaffolds were analyzed regarding their physicochemical properties using scanning electron microscopy (SEM), atomic force microscopy (AFM), and fourier transform infrared microscopy (FTIR). Figure 2a shows the representative SEM images of the PM, PM-C-G, and PM-L-G scaffolds. It was found that electrospun fibers of PM scaffolds exhibit uniform fibrous features without any beads. The average diameter of the fibers (Figure 2b) in the PM scaffold was 360 ± 50 nm. Coating the PM scaffolds with laminin and collagen introduced roughness to the fiber surface. AFM images (Figure 3a) and quantitative analysis of nanofiber scaffolds (Figure 3b) revealed that PM-C-G (1.2 ± 0.069 μm) and PM-L-G (0.9 ± 0.055 μm) had a significantly higher surface roughness compared to that of PM (0.09 ± 0.009 μm) scaffolds. No significant changes were found regarding the average fiber diameter after coating. However, the thickness of the scaffolds after protein coating increased significantly (Figure 2c).

**Figure 4:**
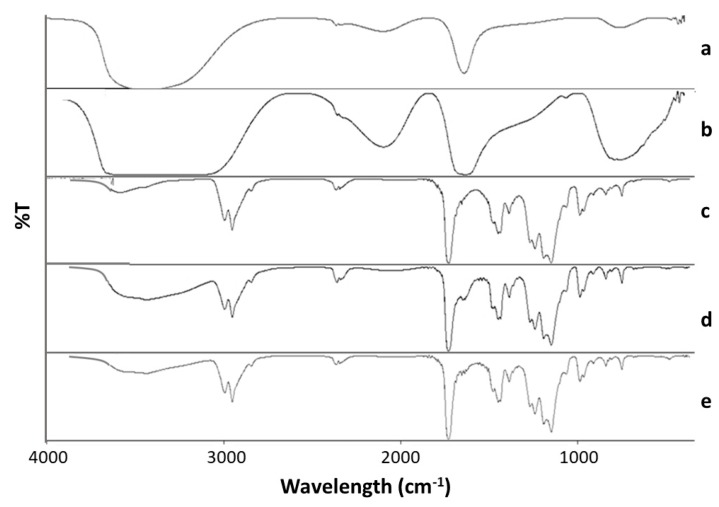
FTIR spectra for (**a**) collagen; (**b**) laminin; (**c**) PM; (**d**) PM-C-G and (**e**) PM-L-G.

Figure 4 shows the IR spectra of the PM, PM-C-G, and PM-L-G scaffolds along with those for collagen, and laminin. The FTIR spectrum of the PM scaffolds shows the presence of characteristic absorption vibrations at 843, 987, and 1062 cm^−1^. In addition, characteristic absorption bands can be seen at 1150, 1444, 1732, and 2952 cm^−1^ for O-CH_3_ stretching bending vibration of the C–H bonds of the –CH_3_ group, acrylate carboxyl group, and C–H bond stretching vibrations, respectively. Collagen and laminin showed characteristic absorptions at ~1639 and 1644 cm^−1^, respectively, which can be assigned as amide I. This is mainly associated with C=O stretching vibrations. The spectra of PM-C-G and PM-L-G contain the absorption bands of both PM and laminin/collagen. The bands indicating the introduction of laminin and collagen can be observed at ~1700–1721 cm^−1^ alongside the PMMA absorption bands.

### 2.3. Mechanical Properties of Nanofiber Scaffolds

**Figure 5:**
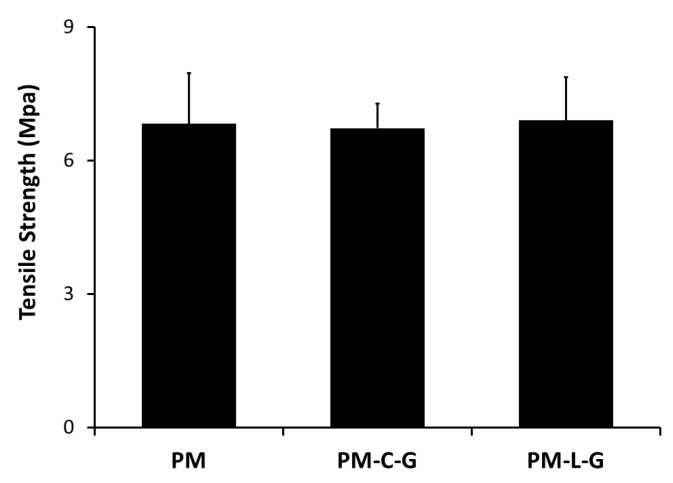
Mechanical properties of PMMA coated and non-coated nanofibers (*n* = 15). Tensile strength tested applying 20 N load until breakage was detected.

Tensile strength was calculated from the stress–strain curve (Figure 5). The tensile strength of the PM scaffold was 6.83 ± 1.13 MPa. Coating the PM scaffolds with collagen or laminin did not affect the tensile strength of the scaffold.

### 2.4. Morphology of Muscle Cells on Nanofiber Scaffolds

**Figure 6:**
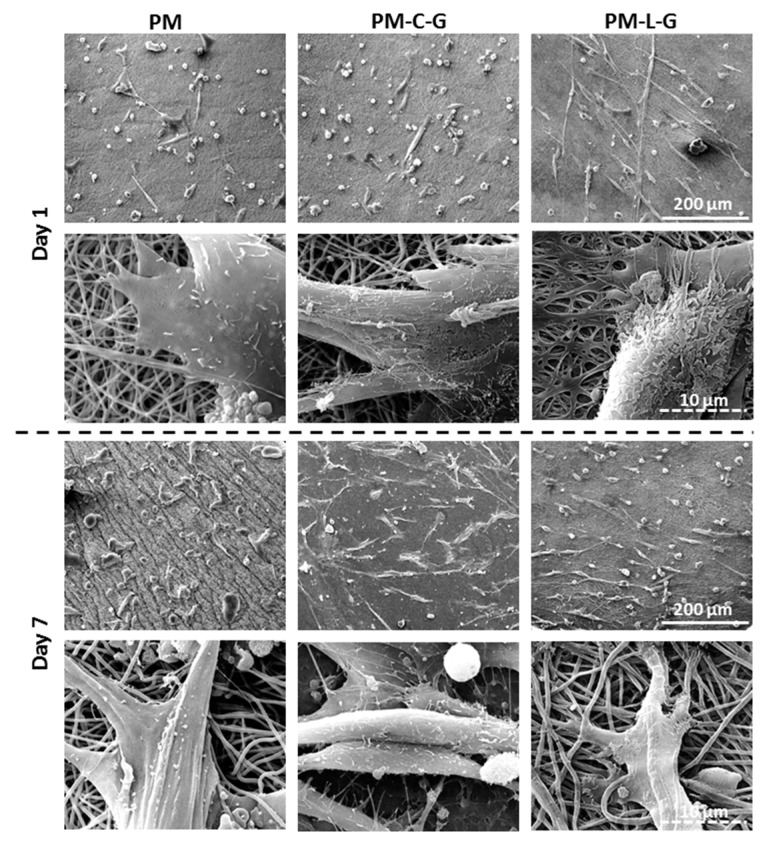
Scanning electron micrographs showing cell morphology at low and high magnification.

Figure 6 shows representative micrographs of muscle cells containing both myoblasts and fibroblasts on PM, PM-C-G, and PM-L-G scaffolds. It was found that, at day 1, cells attached to the PM and PM-C-G scaffolds exhibited a polygonal morphology, whereby cells displayed an elongated morphology on PM-L-G. Cells on PM-L-G demonstrated the formation of filopodia, which were strongly attached to fibers. Culturing the cells until day 7 demonstrated a spread out and flattened morphology of cells on PM-C-G and PM-L-G, whereas cells on the PM surface retained a polygonal shape.

### 2.5. Growth Properties of Muscle Cells

**Figure 7:**
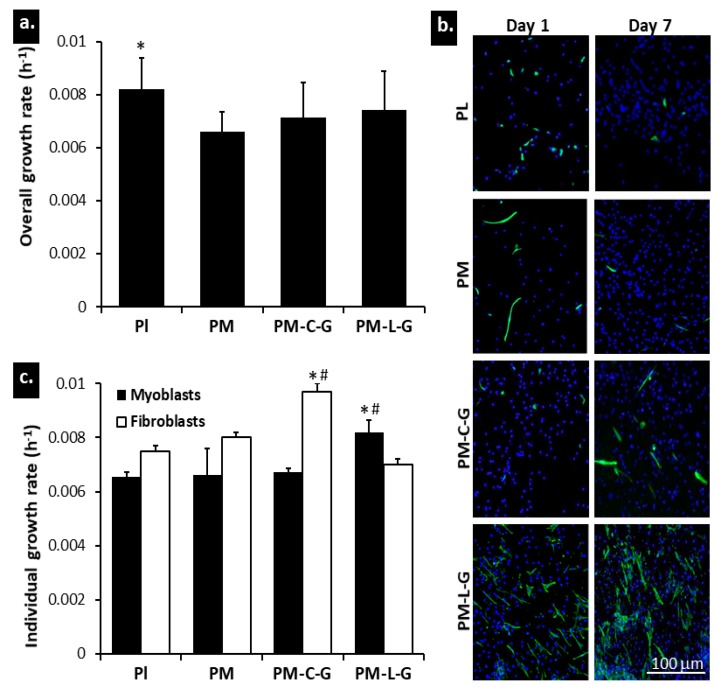
Myoblast and fibroblast (**a**) overall growth rate; (**b**) individual growth rate; and (**c**) cell population at day 1 and day 7. A higher number of cells was found at day 7 compared to day 1. Nuclei are stained with DAPI (blue), and myoblasts are stained with desmin (green). * indicates significantly higher growth rate compared to other culture conditions; # indicates significantly higher growth rate compared to other cell type in same culture conditions.

Mixed populations of myoblasts and fibroblasts were cultured on nanofiber scaffolds and a plastic surface, and the growth rate was evaluated using the Presto Blue assay. As shown in Figure 7a, the overall growth rate of muscle cells on the plastic surface (0.0081 ± 0.0007 h^−1^) was significantly higher than those on the PM, PM-C-G, and PM-L-G scaffolds. No significant differences in overall growth rates were observed among the nanofiber scaffolds. To evaluate the individual growth rate of myoblasts and fibroblasts, muscle cells were stained for desmin to evaluate the number of myoblasts and fibroblasts at days 1 and 7. Figure 7b shows representative fluorescence images of muscle cells under different culture conditions, where desmin positive cells are considered myoblasts and desmin negative cells are considered fibroblasts. It was found that PM-L-G preferentially improved the growth of myoblasts (0.0081 ± 0.0007 h^−1^) (Figure 7c). Conversely, the growth rate of fibroblasts was higher on PL, PM, and PM-C-G. The growth rate of fibroblasts on PM-C-G (0.0097 ± 0.0009 h^−1^) was significantly higher than that of myoblasts and fibroblasts under other culture conditions.

### 2.6. Proliferative Potential of Muscle Cells

**Figure 8:**
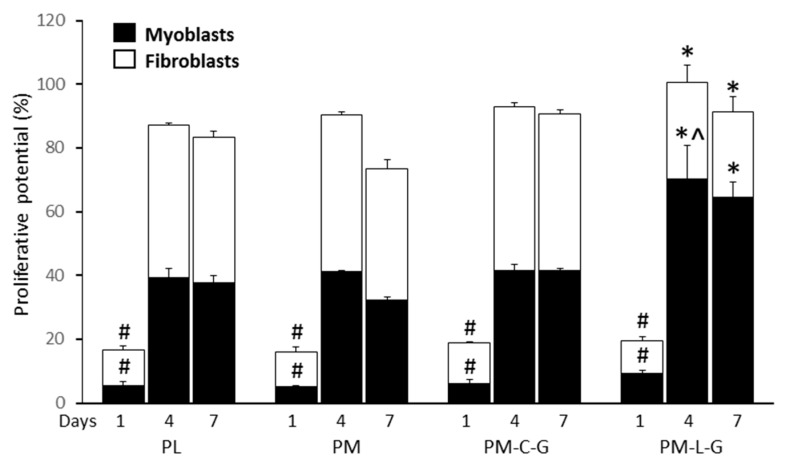
Proliferative potential of fibroblast and myoblasts. # indicates significance difference between groups; * indicates a significant difference between fibroblasts/myoblasts from different culture conditions on the same day; ^ indicates a significant difference between myoblasts in PM-L-G with other culture conditions.

The results of assessing cell proliferation revealed that the number of proliferating fibroblasts and myoblasts in all groups increased significantly over time. The number of proliferating cells in the PM-C-G group was significantly higher for fibroblasts at day 4 (58.2 ± 5.2%) and day 7 (58.1 ± 4.8%) compared to other groups. However, PM-L-G was associated with a significant increase (70.6 ± 10.5%) in proliferating myoblasts compared to other conditions, as shown in Figure 8.

### 2.7. Myoblast Population on Nanofibre Scaffolds

**Figure 9:**
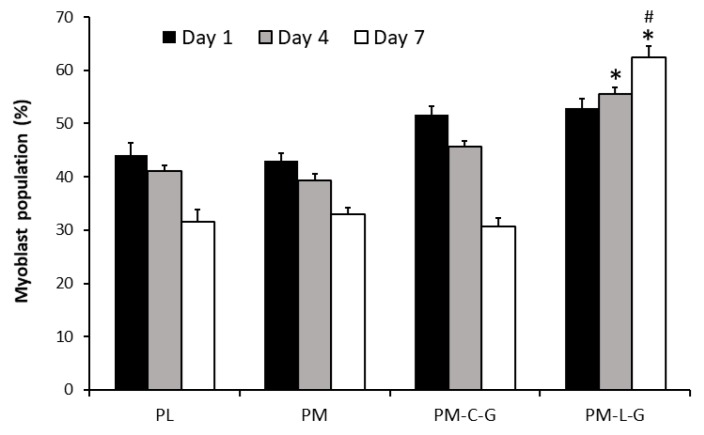
Graph is presenting the percentage of myoblast population. # indicates significance difference between groups; * indicates significance difference of myoblasts population from different culture conditions on the same day.

The percentage of myoblasts under different culture conditions was evaluated at day 1, day 4, and day 7. At day 1, the percentage of myoblasts under different culture conditions was in the range of 43 ± 5% to 51 ± 5%, and the values were not significantly different (Figure 9). However, over time, the percentage of myoblasts decreased on PL, PM, and PM-C-G scaffolds, as the percentage of myoblasts at day 7 was significantly lower than at day 1 for these culture conditions. In the case of PM-L-G, the percentage of myoblasts increased over time, and at day 7 the percentage of the myoblast population (62.35 ± 2.2%) was significantly higher compared to that at day 1. Moreover, the percentage of the myoblast population on PM-L-G at day 7 was significantly higher than other culture conditions.

### 2.8. Cell Migration

**Figure 10:**
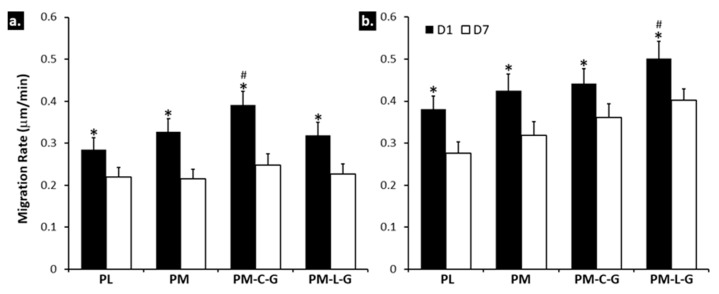
Migration rate of (**a**) fibroblast (**b**) myoblast. # indicates significant difference between groups, * indicates significant difference of fibroblasts/ myoblasts between day 1 and day 7 from same group of culture condition.

The migration rates of myoblasts and fibroblasts under different culture conditions were evaluated by live imaging. It was found that the migration rates of both myoblasts and fibroblasts under different culture conditions decreased significantly at day 7 compared to day 1 (Figure 10a). At day 1, the migration rates of myoblasts and fibroblasts were significantly higher on PM-L-G scaffolds (0.26 ± 0.04 μm/min) and PM-C-G scaffolds (0.23 ± 0.03 μm/min), respectively, compared to other culture conditions. However, no significant differences were observed for the migration rates of myoblasts and fibroblasts among the culture conditions at day 7.

## 3. Discussion

Human skeletal muscle cells containing contractile myoblasts and non-contractile fibroblasts are used in therapeutic applications for several muscle disorders [20]. Enrichment of fibroblasts during culture, due to their higher growth rate, is one of the major challenges encountered while expanding muscle cells and developing engineered tissue substitutes. Several techniques are used for the preferential isolation of myoblasts and the regulation of cell growth (i.e., enhancing myoblast growth and reducing fibroblast growth) such as coating the surface with laminin [21]. However, enrichment of the myoblast population in a 3D culture environment for the development of tissue substitutes has not been investigated. This study was carried out to identify suitable culture conditions for enriching the myoblast population in a 3D culture environment, i.e., electrospun PM nanofiber scaffolds functionalized with ECM proteins such as laminin and collagen. The results suggest that PM scaffolds coated with laminin preferentially enhance the growth and migration of myoblasts, and consequently enrich the myoblast population in the scaffold.

Electrospinning is a simple and cost-effective technique to fabricate long fibers with diameters in the nanometer range. It also offers control over the process parameters to produce nanofiber scaffolds with the desired fiber diameter, morphology, and mechanical properties suitable for specific applications. In this study, PM scaffolds were fabricated using a 5% PMMA solution and flow rate of 1 mL/h for 30 min. The fiber diameter was approximately 360 nm, and the thickness of the scaffold was approximately 0.02 mm. It is well-known that the topographical features of nanofiber scaffolds can regulate the biological properties of cells. However, biofunctionalization of nanofiber scaffolds with functional proteins offers an additional benefit in terms of regulating the tissue-specific functions of cells. Blending synthetic polymers with ECM is commonly employed to introduce bio-functional motifs to the nanofiber scaffold. However, the use of organic solvents presents the risk of denaturing the ECM proteins. Thus, immobilization of ECM proteins by adsorption was employed in this study. A previous study by Rabiatul et al. (2015) demonstrated that the adsorption of collagen on PM scaffolds increases significantly when crosslinked with genipin [22]. Genipin is a natural crosslinker that generates crosslinks spontaneously with ECM proteins such as collagen, gelatin, and chitosan [23]. In this study, PM scaffolds were coated with the most abundant ECM proteins in skeletal muscle tissue, i.e., laminin and collagen. As expected, genipin crosslinking significantly enhanced the adsorption of collagen and laminin on crosslinked PM scaffolds compared to non-crosslinked ones. The higher adsorption of ECM proteins in the presence of genipin could be due to the roughness and wettability of the nanofiber scaffold [24,25], and formation of a stable bond between genipin and ECM proteins [26]. Adsorption of collagen and laminin on PM scaffolds introduced roughness to the nanofiber surface. However, no effect was observed on the fiber diameter.

The mechanical strength of the scaffold plays a crucial role in the development of engineered skeletal muscle tissue. However, the mechanical strength of scaffolds used for tissue engineering applications may not necessarily be the same as that of the native tissue, so it is important to ensure that scaffolds do not fail under physiological conditions [27,28]. The tensile strengths of the PM scaffolds were evaluated at approximately 6.8 MPa, which is similar to that of soft human tissues [29]. No changes in the tensile strength were observed for collagen- and laminin-coated PM scaffolds, although significant increases in scaffold thicknesses were measured.

The influences of nanofiber scaffolds on cell morphology, proliferation, migration, orientation, and differentiation have long been recognized [30,31]. Several studies have demonstrated the superior and faster attachment of cells and the formation of filopodia and lamellipodia during cell attachment [32,33,34]. Moreover, cells also elongate along nanofibers. However, no notable differences in the proliferation and migration of muscle cells were observed on PM scaffolds, which also justifies the importance of biofunctionalization of nanofiber scaffolds for modulating cell properties. A similar observation was reported by Koh et al. (2008) [35], in which they demonstrated that a high concentration of laminin on the surface of poly (l-lactic acid) (PLLA) fibers enhanced cell interactions with scaffolds compared to scaffolds without laminin. Nune et al. (2015) [17] also demonstrated that the immobilization of functional peptides on nanofiber scaffolds significantly increased Schwann cell adhesion and proliferation, and had an impact on the gene expression profile. It was found that biofunctionalization of PM scaffolds with laminin and collagen demonstrates notable changes in muscle cell properties. Muscle cells containing myoblasts and fibroblasts exhibited elongated morphology on ECM-coated PM scaffolds on days 1 and 7 compared to the PM scaffolds, and the elongation of cells was prominent in PM-L-G scaffolds. ECM proteins, including laminin and collagen, are adhesive proteins in tissue that facilitate cellular adhesion through transmembrane integrin receptors. It is well-known that integrin signaling regulates cellular spreading via formation of organized actin fibers, which in turns involves the activation of signal transduction pathways related to proliferation and migration [36]. Although laminin and collagen are major ECM protein component in the skeletal muscle tissue, laminin functions as the main adhesion molecule in the basement membrane. Thus, it was assumed that laminin-coating of PM scaffolds would provide a suitable environment that favors cellular attachment and spreading compared to other scaffolds. Moreover, previous studies have also suggested that laminin favors the attachment of myoblasts over fibroblasts cells, and have used it to separate these two cell types [13,19].

One of the long-standing problems of in vitro myoblast expansion is accompanied with the overgrowth of contaminating fibroblasts. Thus, production of pure myoblasts is still challenging, and limits its uses in muscle development studies and clinical applications [37]. Numerous studies have suggested that surface coating with ECM proteins, especially laminin and collagen type I, enhance the proliferation and differentiation of myoblasts [38,39]. Similar output was also reported for a 3D collagen matrix and a decellularized muscle matrix [40]. However, the majority of those studies were performed using purified myoblast cell lines, and thus, were unable to mimic the practical culture condition containing a mixed population of myoblasts and fibroblasts when cells were acquired from muscle tissue [41,42]. It is well known that laminin enhances the proliferation, migration, and differentiation of myoblasts [20,43,44]. In our previous study, we had demonstrated the preferential effect of laminin on myoblast proliferation compared to its effect on contaminating fibroblasts in a 2D culture condition [19].

In this study, we address the effect of laminin and collagen on regulating myoblast and fibroblast growth and migratory properties during in vitro culture on a 3D environment, i.e., PM scaffolds. Proliferation and migration of cells play important roles in the development and regeneration of damaged tissue. Johnson et al. (2009) [45] demonstrated that electrospun nanofibers provide sufficient adhesion for cells exerted by the cytoskeleton, which promotes cell migration and proliferation. The migration of cells on nanofibers provides more relevant information compared to migration on planar surfaces, due to the structural similarities between nanofiber scaffolds and ECM [46]. Culturing of a mixed population of cells revealed that PM-L-G scaffolds preferentially improve the proliferation and migration of myoblasts, while PM-C-G scaffolds favor the proliferation and migration of fibroblasts. It was noticed that the growth and migration rates of fibroblasts and myoblasts, respectively, on PM-L-G and PM-C-G were not affected, indicating the cell-specific stimulation by laminin and collagen. It has been well documented, in 2D culture conditions, that laminin-coated surfaces enhance the growth of myoblasts by increasing cell division, maintaining higher number of proliferative cells, and reducing cellular fusion and the formation of terminally differentiated myotubes [20,44,47]. Enhancement of myoblast migration on the laminin-coated surface was shown to reduce cell-cell contact, which is a prerequisite for initiation of myoblast fusion to form myotubes [20]. Migration of myoblasts on the laminin-coated surface is regulated by the integrin α7β1 receptor [47]. The β1 domain of the integrin receptor is involved in the enhancement of myoblast migration by increasing the formation of focal adhesion and stress fibers [48]. Moreover, β1 is also associated with the growth factor receptor and downstream mitogen-activated protein kinase (MAPK), which facilitates cellular proliferation [49]. It was also postulated that the epidermal growth factor (EGF)-like motif on laminin might also act as a mitogenic activator for myoblasts [50]. In contrast, most of cells bind to collagen mainly by α2β1 or α3β1 integrin receptors [51]. Since the fibroblasts naturally produce ECM fibers made of collagen and elastin, collagen coating could provide an appropriate substrate for the attachment and migration of fibroblasts rather than myoblasts. The increase in myoblast proliferation on PM-L-G scaffolds resulted in enrichment of the myoblast population throughout the culture of seven days. In contrast, the myoblast population decrease on PM and PM-C-G scaffolds. It was also observed that the migration of myoblasts and fibroblasts decreased over time. This could be due to the loss of vacant space on the surface due to the higher confluence.

## 4. Materials and Methods

### 4.1. Fabrication of PMMA Nanofiber (PM) Scaffolds

A 5% PM (340,000 *m*/*w*; Sigma Aldrich, St. Louis, MO, USA) solution (*w*/*v*), dissolved in hexa-fluoro-2-propanol (HFIP; Sigma Aldrich), was used for electrospinning. The freshly prepared polymer solution was loaded into a 1 ml syringe equipped with a 21-gauge blunt needle, and placed in a syringe pump. The rotating drum collector was placed at a 10-cm distance from the needle tip. To fabricate PM scaffolds, the solution was pumped at a rate of 1 mL/h for 30 min with an applied voltage of ± 10 kV and the rotation speed of the collector set at ~2800 rpm. PM scaffolds were sterilized overnight under ultraviolet (UV) light in a biosafety cabinet and washed with Dulbecco’s phosphate buffered saline (DPBS; Gibco, Gaithersburg, MD, USA) prior to the experiment.

### 4.2. Laminin (L) and Collagen (C) Coating

PM scaffolds were coated with Engelberth–Holm–Swarm murine sarcoma laminin-1 (L; Sigma Aldrich, USA) and rat tail collagen type 1 (C; Sigma Aldrich) as described elsewhere [16,22]. In brief, PM scaffolds were incubated with laminin-1 (50 μg/mL) and collagen type I (50 μg/mL) solutions for 2 h at 37 °C, followed by washing DPBS to prepare PM-L and PM-C scaffolds, respectively. Furthermore, PM scaffolds were coated with laminin-1 and collagen type I in the presence of the crosslinker agent genipin (G; Wako Chemicals, Osaka, Japan). The laminin-1 (50 μg/mL) and collagen type I (50 μg/mL) solutions were mixed with 0.002% genipin before being added to the PM scaffolds and incubated for 2 h at 37 °C to prepare PM-L-G and PM-C-G scaffolds, respectively.

### 4.3. Protein Quantification Assay

PM, PM-L, PM-C, PM-L-G, and PM-C-G scaffolds were cut into circular pieces (34.8 mm in diameter) and weighed prior to being placed in 6-well plate. The bicinchoninic assay (BCA, Sigma Aldrich) was performed to evaluate the amount of protein adsorbed on the scaffolds according to the protocol described by Rabiatul et al. (2015) [22]. In brief, all the scaffolds were incubated with the BCA working reagent for 30 min at 37 °C, and absorbance was measured at 562 nm. The concentration of protein was measured using the standard curve of the bovine serum albumin protein standard.

### 4.4. Scanning Electron Microscopy

The ultrastructure of the nanofiber scaffolds was observed via scanning electron microscopy (SEM). In brief, nanofiber scaffolds were fixed with 2% glutaraldehyde (GA) for 24 h at 2–8 °C, followed by multiple washes with DPBS. The samples were then dehydrated with increasing concentrations of alcohol. The dehydrated samples were then dried in a critical point dryer (Baltec 030 CPD, Liechtenstein, Switzerland) and sputter coated (Polaron E5100 sputter coater, Milan, Italy) with gold. The samples were then observed using SEM. SEM images were used to evaluate fiber diameter. At least 90 fibers from each scaffold were chosen randomly, and the diameter of the fiber was measured using the integrated software of the SEM equipment.

### 4.5. Atomic Force Microscopy (AFM)

The surface roughness of the nanofiber scaffold was evaluated using a tapping mode AFM (NTEGRA Prima/NT-MDT, Moscow, Russia). The root-mean-square roughness (Rq) values determined the mean roughness of the nanofiber’s sheet. Forces between a sharp probe were measured to determine a 3D topographic structure of the scaffold by probing its surface. Three samples were evaluated for each group. Each image was then processed using Image Analysis P9 (version 3.5.0.2068, Moscow, Russia). All samples were scanned at room temperature.

### 4.6. Mechanical Properties

Tensile testing of nanofiber scaffolds was carried out using a 20 N load cell (Model UUK 5, Seoul, Korea) equipped with an Ezi Step micro stopper motor system (Fastec, Seoul, Korea). All samples were tested in wet conditions to mimic the actual culture conditions in vitro. A total of 15 samples were tested for each scaffold. The test involved applying tensile forces on a test specimen whose axis was perpendicular to the grips. Static tests were conducted by inserting nanofiber scaffolds into the gripped part of the specimen to prevent crushing. The thickness of the sample was evaluated using an absolute digimatic indicator (Mitutoyo, Takatsu-ku, Japan). At least three random points on each sample were measured to evaluate the average thickness of the scaffold. The values of tensile strength were obtained from the stress and strains curve.

### 4.7. Fourier Transform Infrared (FTIR) Spectroscopy

To characterize the chemical composition of the nanofibre scaffolds, FTIR was performed using Spectrum GX FTIR spectrometer (Perkin Elmer, Waltham, MA, USA). The samples were scanned in the range of 4000–500 cm^−1^. In total, three samples from each scaffold were assessed.

### 4.8. Skeletal Muscle Cells Isolation and Primary Culture

Human skeletal muscle samples were collected as redundant tissues from patients undergoing amputation. Written consent was obtained from all patients prior to surgery. Prior to commencing the study, ethical approval was obtained from the Research and Ethical Committee of Universiti Kebangsaan Malaysia with a reference number, 02-01-02-SF1284. 

To begin the process, skeletal muscle tissues were cleared of fat, connective tissue, and blood vessels. The sample was then minced into small pieces and digested with 0.25% trypsin-EDTA (TE, Gibco) for 30 min. TE was then neutralized with fresh culture medium containing 10% fetal bovine serum (FBS; Gibco). The digestion cycle was repeated at least four times. The cells were cultured and maintained in the equivalent mixture of high glucose F10 (Sigma Aldrich, USA) and Dulbecco’s Modified Eagle Medium (DMEM) containing 10% FBS at 37 °C under a 5% CO_2_ atmosphere. The medium was replaced every 2 days, and cells were trypsinized at 80% confluence. The culture was maintained until passage (P) 3. To evaluate the cellular properties of nanofiber scaffolds, cells at P4 were seeded on PM, PM-C-G, and PM-L-G at a seeding density of 6 × 10^3^ viable cells/cm^2^. Cells cultured on the plastic surface (PL) were used as the control.

### 4.9. Viability Assay

The viability of cells on PL, PM, PM-C-G, and PM-L-G, were evaluated by the Presto Blue assay kit (Thermo Fisher Scientific, Waltham, MA, USA). The evaluation was performed at days 1, 4, and 7 after seeding. Briefly, waste medium from the culture plate was replaced with 10% presto blue solution prepared in culture medium and incubated for 2 h at 37 °C. The viability of the cells was evaluated by measuring the absorbance (OD) value at 570 nm using a spectrophotometer.

### 4.10. Immunofluorescence Staining (ICC)

ICC was carried out to evaluate the proportions of myoblasts and fibroblasts over time under different culture conditions. The growth rate and proliferative potential of myoblasts and fibroblasts were also evaluated via ICC. The antibodies, their specificity and dilution factors are shown in Table 1 and the equations used to evaluate the proportion of myoblasts and fibroblasts, their growth rate and proliferative potential are shown in Table 2. Briefly, cells were fixed with 4% paraformaldehyde (Sigma Aldrich), permeabilized with 0.05% Triton X-100 (Sigma Aldrich) and blocked with 10% goat serum (Sigma Aldrich). Cells were then incubated with primary antibody, followed by secondary antibody. Cells were then counterstained with DAPI and observed under a confocal laser scanning microscope (CLSM; Nikon, Tokyo, Japan). At least five images were captured randomly from each culture well to evaluate total cells, myoblasts, and fibroblasts on days 1, day 4, and 7 after seeding. Three technical replicates were carried out for each sample (*n* = 3).

### 4.11. Cell Migration

Time-lapse imaging was performed to determine the migration rates of myoblasts and fibroblasts at day 1 and day 7 using a CLSM equipped with an automated stage and a Chamlide incubation system (Live Cell Instrument, Seoul, Korea). To improve the visibility of cells during time-lapse imaging, a thin layer of the nanofiber scaffold was prepared. The phase contrast images were captured every 20 min for 24 h. Cells were fixed immediately and stained for desmin to identify myoblasts according to the protocol described earlier. Desmin negative cells were identified as fibroblasts. A quantitative evaluation of the migration rate was performed by measuring the distance traveled by the cells for 1 h at an interval of every 20 min. Three technical replicates were carried out for each sample (*n* = 3).

### 4.12. Statistical Analysis

All the results are expressed as means ± standard deviation (SD). One-way analysis variance (ANOVA) was used in this study to test for significant differences; *p* < 0.05 was considered statistically significant.

## 5. Conclusions

In conclusion, we successfully fabricated PM nanofiber scaffolds with a fiber diameter of approximately 360 nm and a mechanical strength similar to that of human soft tissue. The most abundant ECM proteins in skeletal muscle, i.e., laminin and collagen type I, were immobilized on the scaffolds via adsorption, which did not affect fiber diameter and mechanical strength. It was also found that coating PMMA nanofiber scaffolds with laminin preferentially enhanced the proliferation and migration of myoblasts, which allowed for the enrichment of myoblasts from approximately 51% to 62% over seven days of culture. In contrast, coating PM nanofiber scaffolds with collagen enhanced the proliferation and migration of fibroblasts, thus, significantly reducing the myoblasts population from approximately 51% down to 31%. These results confirm the specific roles of laminin and collagen in regulating the biological properties of myoblasts and fibroblasts. Based on these results, we can summarize that laminin-coated nanofiber scaffolds are suitable for developing skeletal muscle tissue substitutes, which can be used for clinical applications, as well as an in in vitro 3D models to study muscle development, and for drug discovery.

## Figures and Tables

**Table 1:** ICC protocol.

1° Antibody/Counterstain	Specificity	2° Antibody
Anti-mouse Ki67 (1:250; Abcam, Cambridge, UK)	Nuclear protein expressed during proliferation	Alexa Fluor Goat anti-mouse 594 (1:300; Invitrogen, Carlsbad, CA, USA)
Anti-rabbit desmin (1:300; Novusbio, Littleton, CO, USA)	Myoblast	Alexa Fluor Goat anti-mouse 488 (1:300; Invitrogen, USA)
4,6-diamidino-2-phenylindole (1:15,000; DAPI; Molecular Probes, Eugene, OR, USA)	Nuclei	Not applicable

**Table 2:** The equation to evaluate proportion of myoblasts and fibroblasts, growth rate and proliferative potential.

Assay	Staining of Cells	Equation
Proliferative potential	Desmin (Myoblasts) + Ki67 (Proliferating cells) + DAPI (Nucleus) *[Desmin negative cells were identified as fibroblasts]*	$\frac{Total\;proliferating\;myoblasts\;or\;fibroblasts\;\times 100}{Total\;cells\;\left(DAPI\;positive\right)}$
Growth rate of myoblasts and fibroblasts	Desmin (Myoblasts) + DAPI (Nucleus) *[Desmin negative cells were identified as fibroblasts]*	Growth rate (h^−1^) = ln (Xa_2_/Xa_1_)/∆t
Percentage of myoblasts/fibroblasts	Desmin (Myoblasts) + DAPI (Nucleus) *[Desmin negative cells were identified as fibroblasts]*	$\frac{Total\;myoblasts/fibroblasts}{Total\;cells}\times 100$

## Abbreviations

ANOVA	One way Analysis Variance
AFM	Atomic Force Microscopy
BCA	Bichincionic Assay
C	Collagen
CPD	Critical Point Dryer
CLSM	Confocal Laser Scanning Microscope
D	Day
DAPI	4,6-Diamidino-2-phenylindole
DMEM	Dulbecco’s Modified Eagle’s Medium
DPBS	Dulbecco’s Phosphate Buffered Saline
ECM	Extracellular Matrix
FBS	Fetal Bovine Serum
Fig.	Figure
FTIR	Fourier Transform Infrared
G	Genipin
GA	Glutaraldehyde
H	Hour
HFIP	Hexafluoroisopropanol
ICC	Immunofluorescence Staining
L	Laminin
Min	Minute
P	Passage
PL	Plain
PMMA/PM	Polymethl-Metachlaryte
PM-C-G	PMMA Coated with Collagen; Crosslinked with Genipin
PM-L-G	PMMA Coated with Laminin; Crosslinked with Genipin
SEM	Scanning Electron Microscope
SD	Standard Deviations
TE	Trypsin-EDTA
UKM	Universiti Kebangsaan Malaysia
UV	Ultraviolet
USA	United State of America
3D	Three Dimensional
